# Intestinal Lipid Metabolism Genes Regulated by miRNAs

**DOI:** 10.3389/fgene.2020.00707

**Published:** 2020-07-10

**Authors:** María Belén Ruiz-Roso, Judit Gil-Zamorano, María Carmen López de las Hazas, Joao Tomé-Carneiro, María Carmen Crespo, María Jesús Latasa, Olivier Briand, Daniel Sánchez-López, Ana I. Ortiz, Francesco Visioli, J. Alfredo Martínez, Alberto Dávalos

**Affiliations:** ^1^Laboratory of Epigenetics of Lipid Metabolism, Madrid Institute for Advanced Studies (IMDEA)-Food, CEI UAM + CSIC, Madrid, Spain; ^2^Laboratory of Functional Foods, Madrid Institute for Advanced Studies (IMDEA)-Food, CEI UAM + CSIC, Madrid, Spain; ^3^Research Program, Innovation, Communication and Education Program, Madrid Institute for Advanced Studies (IMDEA)-Food, CEI UAM + CSIC, Madrid, Spain; ^4^University of Lille, Inserm, Centre Hospitalier Universitaire (CHU) de Lille, Institut Pasteur de Lille, U1011-European Genomic Institute for Diabetes, Lille, France; ^5^Servicio de Cirugía Experimental, Hospital Universitario Ramón y Cajal, IRYCIS, Madrid, Spain; ^6^Department of Molecular Medicine, University of Padua, Padua, Italy; ^7^Department of Nutrition and Physiology, Center for Nutrition Research, University of Navarra, IDISNA Navarra, Pamplona, Spain; ^8^Centre of Biomedical Research in Physiopathology of Obesity and Nutrition (CIBEROBN), Institute of Health Carlos III, Madrid, Spain; ^9^Cardiometabolic Nutrition Group, Madrid Institute for Advanced Studies (IMDEA)-Food, CEI UAM + CSIC, Madrid, Spain

**Keywords:** Dicer1, lipid metabolism, small intestine, microRNA, organoids, *Hmgcs2*, *Acat1*, *Olr1*

## Abstract

MicroRNAs (miRNAs) crucial roles in translation repression and post-transcriptional adjustments contribute to regulate intestinal lipid metabolism. Even though their actions in different metabolic tissues have been elucidated, their intestinal activity is yet unclear. We aimed to investigate intestinal miRNA-regulated lipid metabolism-related genes, by creating an intestinal-specific Dicer1 knockout (Int-Dicer1 KO) mouse model, with a depletion of microRNAs in enterocytes. The levels of 83 cholesterol and lipoprotein metabolism-related genes were assessed in the intestinal mucosa of Int-Dicer1 KO and Wild Type C57BL/6 (WT) littermates mice at baseline and 2 h after an oral lipid challenge. Among the 18 genes selected for further validation, *Hmgcs2*, *Acat1* and *Olr1* were found to be strong candidates to be modulated by miRNAs in enterocytes and intestinal organoids. Moreover, we report that intestinal miRNAs contribute to the regulation of intestinal epithelial differentiation. Twenty-nine common miRNAs found in the intestines were analyzed for their potential to target any of the three candidate genes found and validated by miRNA-transfection assays in Caco-2 cells. MiR-31-5p, miR-99b-5p, miR-200a-5p, miR-200b-5p and miR-425-5p are major regulators of these lipid metabolism-related genes. Our data provide new evidence on the potential of intestinal miRNAs as therapeutic targets in lipid metabolism-associated pathologies.

## Introduction

Lipid metabolism consists of anabolic and catabolic processes, in which the intestinal epithelium plays a very important role in maintaining systemic energy homeostasis (Zhou et al., 2020). Lipid absorption in the gastrointestinal tract is essentially carried out by the enterocytes, where digested lipids are packed into chylomicrons and secreted to circulation, through which they reach distal tissues (Desmarchelier et al., 2019). The whole process involves considerable adjustments concerning morphological, transcriptional and posttranscriptional responses. Evidence shows that there is an increase in the uptake of fatty acids (FA) and cholesterol by the small intestine, which affects lipid metabolism gene and protein expression, in mice fed high-fat diets (HFD) (de Las Heras et al., 2017). Even though all cellular processes behind intestinal lipid metabolism are not fully deciphered, microRNAs (miRNAs), which are important posttranscriptional gene regulators (Sand, 2014; Lopez de Las Hazas et al., 2019), have been investigated in enterocytes (Gil-Zamorano et al., 2014). Indeed, miRNAs participate in processes of intestinal epithelial differentiation (Dalmasso et al., 2010) and barrier function (Ye et al., 2011). Moreover, miRNAs modulate the expression of certain lipids, cholesterol, and FA synthesis and metabolism-related genes (Gil-Zamorano et al., 2014), like 3-*Hydroxy-3-methylglutaryl coenzyme A synthase (Hmgcs)*, *Acetyl-CoA acetyltransferase (Acat)*, *fatty acid synthase (Fasn)*, *ATP-Binding Cassette Transporter A (Abca)*, *C-Protein reactive (Crp) or Scavenger receptor class B type 1* (Briand et al., 2016; Davalos-Salas et al., 2019). Yet, it is likely that many other miRNA-regulated genes involved in intestinal lipid metabolism remain unknown. Gain- and loss-of function studies showed that miRNA dysregulation may not be critical in normal tissues but can greatly affect the performance of cells and tissues undergoing stress conditions (Mendell and Olson, 2012). In this sense, the expression of miRNAs in the intestinal epithelium becomes dysregulated in several diseases, such as in various types of cancer (Ahmed et al., 2018), inflammatory bowel disease (Feng et al., 2019), necrotizing enterocolitis (Ng et al., 2015), and diabetes mellitus (Shan et al., 2016).

MiRNAs are small (19–25 nucleotides) non-coding RNA molecules, which modulate the activity of hundreds of genes and different pathways related to key biological processes in enterocytes, such as differentiation, proliferation and apoptosis (Bartel, 2009; Gadecka and Bielak-Zmijewska, 2019). Small intestine miRNAs are likely engaged in the regulation of processes such as energy homeostasis, lipid metabolism and HFD-induced weight increase (Briand et al., 2016; Mantilla-Escalante et al., 2019). MiRNAs generally repress the expression of target-genes in lipid metabolism-related pathways, such as insulin signaling, ketogenesis and homeostasis of cholesterol (Li et al., 2020; Wu et al., 2020). Nonetheless, the role of miRNAs concerning the regulation of lipid metabolism in the intestinal epithelium has not been fully investigated.

Proper miRNA production and function requires a complex machinery (Mendell and Olson, 2012; Sand, 2014). A vital element of the miRNA machinery is DICER1, a cytoplasmic RNase III type endonuclease necessary for the biosynthesis of miRNAs and small interfering RNAs (siRNAs). *Dicer1* depletion results in the build-up of miRNA precursors and in decreased levels of mature miRNAs (Huang et al., 2012; Robertson et al., 2018), which becomes useful when studying miRNAs functions.

Loss of *Dicer 1* in the intestinal epithelium has been previously established in intestinal-specific *Dicer1* knockout (Int-*Dicer1* KO) mouse models, showing that depletion of microRNAs in enterocytes disturbs mouse intestinal crypts structure (McKenna et al., 2010), lipid metabolism (Mantilla-Escalante et al., 2019) and intestinal epithelial differentiation (McKenna et al., 2010). Here, the aim was to investigate the expression of miRNA-regulated cholesterol and lipoprotein metabolism-related genes and proteins involved in the homeostatic regulatory machinery of postprandial lipemia, and, in doing so, identifying potential novel therapeutic targets in lipid metabolism disorders.

## Results and Discussion

### Lipid Metabolism Genes Modulated by the Loss of Intestinal *Dicer*1

**FIGURE 1:**
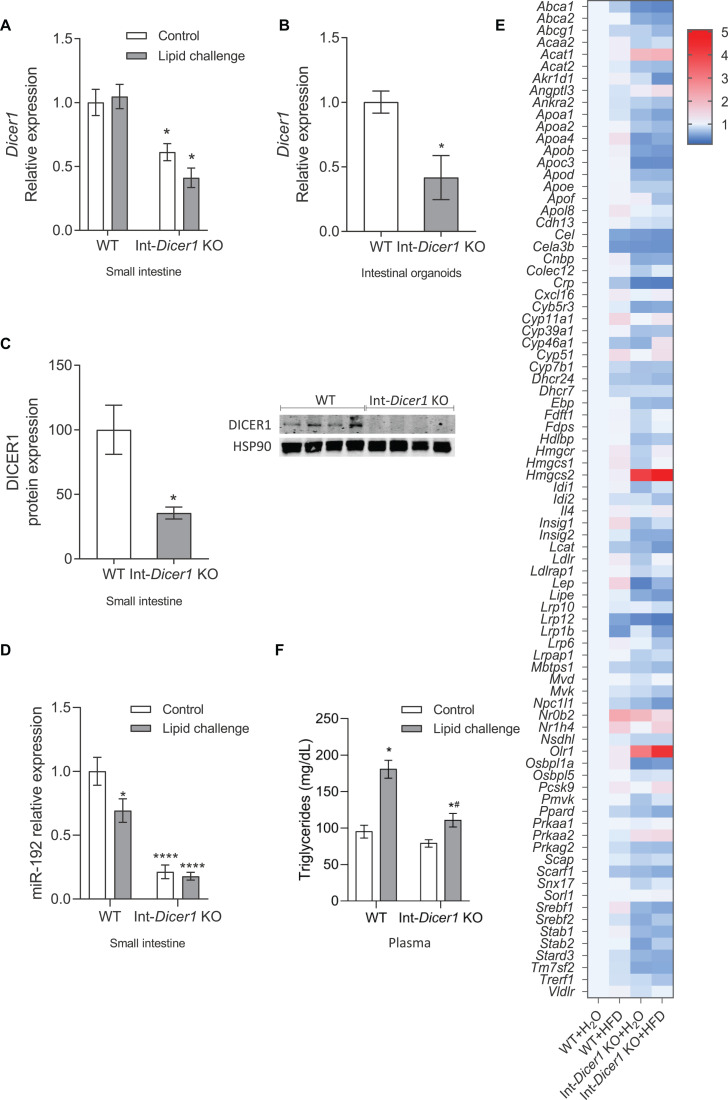
**(A)** Effects of an oral high-fat dietary challenge (lipid challenge) on the gene expression of *Dicer1* in small intestine of Wild Type C57BL/6 (WT) and intestinal-specific *Dicer1* knockout (Int-*Dicer1* KO) mice. *p < 0.05 compared to WT+H_2_O mice (*n* ≥ 23). **(B)** *Dicer1* gene expression in intestinal organoids isolated from WT and Int-*Dicer1* KO mice. *p < 0.05 compared to WT (*n* = 6). **(C)** DICER1 protein expression in small intestine scraped mucosa and intestinal organoids of WT and Int-*Dicer1* KO mice. *p < 0.05 compared to WT group (*n* = 4). **(D)** Intestinal levels of miR-192 in WT and Int-Dicer1 KO mice. *p < 0.05 compared to WT+H_2_O mice (*n* ≥ 23). **(E)** Effects of an oral lipid challenge on the expression of lipid and cholesterol metabolism-related genes, in small intestine from WT and Int-*Dicer1* KO mice. Upregulated genes are represented in red; downregulated genes are shown in blue. **(F)** Effect of an oral lipid challenge on plasma triglyceride levels in WT and Int-Dicer1 KO mice. *p < 0.05 compared to WT+H_2_O, ^#^*p* < 0.05 compared to WT+lipid challenge mice (*n* ≥ 23). Data in panels **(A–D,F)** are means ± SEM. Comparison between groups by two-way ANOVA **(A,F)** or two-tailed unpaired *t*-tests **(B–D)**.

The intestine is considered as the “gatekeeper” of intestinal lipid absorption (Krieger, 2001) and miRNAs are relevant players in lipid metabolism (Briand et al., 2016; Mantilla-Escalante et al., 2019), with peculiar roles under stress conditions (Small and Olson, 2011). Having these two concepts in mind, a mouse model lacking miRNAs in epithelial intestinal cells was created by backcrossing *Dicer1^loxP^/^loxP^* (The Jackson Laboratories, Bar Harbor, ME, United States) and *Villin-cre* mice (The Jackson Laboratories), showing a remarkable reduction of *Dicer1* gene expression in the small intestine and intestinal organoids compared to WT mice (Figures 1A,B). To search for genes directly modulated by miRNAs in response to a lipid challenge, Int-*Dicer1* KO mice were exposed to a single gavage of olive oil + cholesterol (lipid challenge) or water (controls), for 2 h (*n* = 5 per group). Compared to controls, *Dicer1* gene expression did not change after the lipid challenge. As expected, loss of *Dicer1* in cells expressing *Villin1* (*Vill1*) dramatically reduced the levels of DICER1 protein in the small intestine scraped mucosa from Int-*Dicer1* KO mice compared to WT (Figure 1C). Reduced DICER1 protein levels were accompanied by a considerable reduction of miR-192 (Figure 1D), one of the most expressed miRNA in the small intestines (Huang et al., 2012).

Next, the response of 83 genes directly related to cholesterol and lipid metabolism was evaluated by RT-qPCR (Figure 1E). After the lipid challenge, the expression of several genes (*Cyp51*, *Hmgcs1*, *Hmgcs2*, *insulin-induced gene (Insig)*, *LDL receptor (Ldlr)*, *Leptin (Lep)*, *nuclear receptor subfamily 1*, *group H*, *member 4 (Nr1h4)* and proprotein convertase subtilisin/kexin type 9 (*Pcsk9)*) was induced in WT and Int-*Dicer1* KO mice compared with controls. Moreover, the expression of *Abca1*, *Crp*, *low-density lipoprotein receptor-related protein-12 (Lrp12)*, *Lrp1b* and *Niemann-Pick C1 like 1 (Npc1l1)* was repressed, suggesting an appropriate response to dietary lipids. Besides causing detrimental effects on cardiovascular disease, fat-rich diets are linked to gastrointestinal transit disorders, although the mechanisms responsible for these actions are not entirely clear (Nezami et al., 2014). As many genes related to lipoprotein metabolism were differentially expressed in the Int-*Dicer1* KO mice, we next evaluated whether this had a physiological consequence in plasma lipid levels. We found that, after the lipid challenge, plasmatic postprandial triglycerides levels were reduced in Int-*Dicer1* KO mice compared to WT (Figure 1F). This effect was not observed at baseline levels.

**FIGURE 2:**
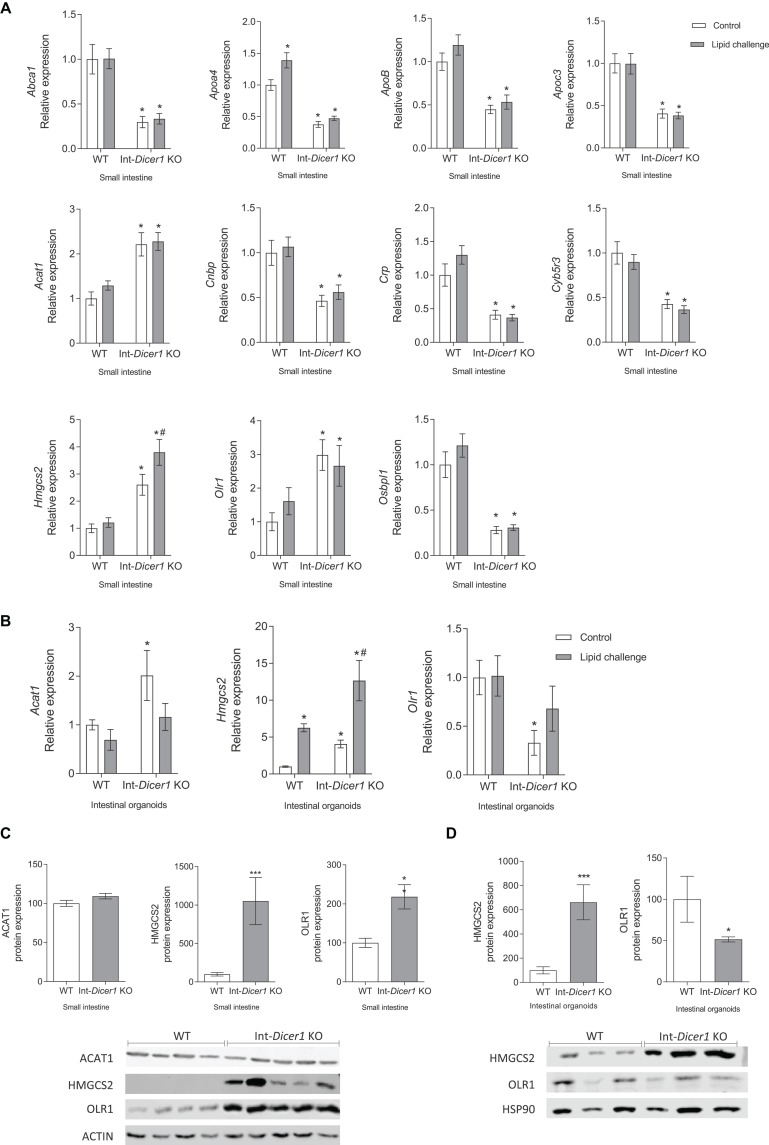
**(A)** Effect of an oral high-fat dietary challenge (lipid challenge) on the expression of *Abca1*, *Apoa4*, *ApoB*, *Apoc3*, *Acat1*, *Cnbp*, *Crp*, *Cyb5r3*, *Hmgcs2*, *Olr1* and *Osbpl1*, in small intestine of Wild Type C57BL/6 (WT) and intestinal-specific *Dicer1* knockout (Int-*Dicer1* KO) mice. *p < 0.05 compared to WT+H_2_O mice, ^#^*p* < 0.05 compared to Int-*Dicer1* KO+H_2_O (*n ≥* 23). **(B)** *Acat1*, *Hmgcs2* and *Olr1* gene expression in intestinal organoids isolated from WT and Int-*Dicer1* KO mice exposed to postprandial micelles of olive oil and cholesterol (PPM). *p < 0.05 compared to control WT mice, ^#^*p* < 0.05 compared to control Int-*Dicer1* KO group (*n* = 9). **(C)** ACAT1, HMGCS2 and OLR1 protein expression in small intestine of WT and Int-*Dicer1* KO mice. **p < 0.001, ***p < 0.0001, compared to WT (*n* = 9). **(D)** HMGCS2 and OLR1 protein expression in intestinal organoids isolated from WT and Int-*Dicer1* KO mice. *p < 0.05, ***p < 0.0001 compared to WT (*n* = 3). Data in all cases are means ± SEM. Comparison between groups by two-way ANOVA **(A,B)** or two-tailed unpaired *t*-tests **(C,D)**.

The hypothesis here was that, in the absence of *Dicer1*, genes whose expression is modulated by miRNAs would show upregulated levels or be differentially modulated in the absence of *Dicer1*. Thus, attention was set on genes whose expression increased in *Dicer1* KO mice compared to WT. Since their expression levels differed in Int-*Dicer1* KO mice compared to WT, we found 18 candidate genes of modulation by miRNAs, namely *Abca1*, *Abca2*, *apolipoprotein A4 (Apoa4)*, *Apoc3*, *Apob*, *Acat1*, *nuclear envelope-enriched activator of lipin (Cnep)*, *cytochrome b5 reductase 3 (Cyb5r3)*, *protein kinase AMP-activated α catalytic subunit (Prkaa)*, *orphan receptor small heterodimer partner (Nr0b2)*, *hormone-sensitive lipase (Lipe)*, *steroidogenic acute regulatory domain 3 (Stard3)*, *Hmgcs2*, *oxidized low-density lipoprotein receptor 1 (Olr1)* and a oxysterol-binding protein (*Osbpl1a)*. Although some of the above-mentioned genes, e.g., *Lipe* showed no changes or very low expression levels (Supplementary Figure S1A), 11 genes were more likely to be modulated by miRNAs, according to the validation assays performed in the small intestine of two different animal cohorts (*n* ≥ 23 per group) (Figure 2A). Interestingly, among these genes, three were strong candidates for modulation by miRNAs in enterocytes and intestinal organoids, namely mitochondrial 3-Hydroxy-3-methylglutaryl coenzyme A synthase (*Hmgcs2*), acetyl-CoA acetyltransferase 1 (*Acat1*) and oxidized low-density lipoprotein receptor 1 (*Olr1*) (Figure 2A). These new potential miRNA-modulated genes have not been reported in previous studies and, together with other previously identified targets (Huang et al., 2012), provide additional evidence of the relevance of miRNAs in lipid metabolism.

### *Hmgcs2* and *ACAT1* Are Modulated by Intestinal miRNAs

Because the canonical function of miRNAs is to repress their target genes, focus was set on two candidate genes of modulation by miRNAs, i.e., *Hmcgs2* and *Acat1*, whose mRNA expression was increased in *Dicer1* KO mice. Here, *Hmgcs2* gene expression was seen to increase in small intestine and intestinal organoids of Int-*Dicer1* KO mice compared to WT, and to rise after the fat challenge (Figures 2A,B). Furthermore, HMCGS2 protein levels were also increased in the small intestine (Figure 2C) and in intestinal organoids (Figure 2D) of Int-*Dicer1* KO mice compared to WT. HMGCS2 catalyzes the first reaction of ketogenesis, or the formation of ketone bodies, in mitochondria of hepatocytes and gut epithelial cells, condensing acetyl-CoA with acetoacetyl-CoA to form methylglutaryl coenzyme A (HMG-CoA) (Puchalska and Crawford, 2017; Kim et al., 2019). The intestinal ability to oxidize fat and generate ketone bodies (a valuable energy resource in fasting cells) has been reported in many studies (Puchalska and Crawford, 2017). Fasting and intense lipolysis are, among other factors, ketogenesis inducers (Hegardt, 1999) and increased expression of HMGCS2 is related to increased ketogenesis in intestinal mucosa (Wang et al., 2017). Furthermore, ketone bodies are markers of mitochondrial dysfunction (Kennaway et al., 1984; Robinson et al., 1985), which can be intensified by high free cholesterol levels (Campbell and Chan, 2008). Here, mice received a single lipid challenge 2 h before sacrifice, rather than being fed a high-fat diet and this could result in a punctual increase in fat consumption at the expense of carbohydrates, promoting a situation of ketogenesis or the formation of ketone bodies. The most striking result we observed is that the absence of *Dicer1* in enterocytes leads to a significant increase in both gene and protein expression of HMGCS2, in small intestine and organoids. Therefore, it is conceivable that, in the small intestine, the physiological process of ketogenesis is highly regulated by miRNAs and that a deficiency in these molecules could affect it. Other genes involved in the metabolism of ketone bodies were also evaluated (i.e., *Bdh1* and *Hmgcl*, Supplementary Figures S1A,B, respectively). The induction of *Bdh1*in the intestines of Int-*Dicer1* KO mice compared to their WT littermates was statistically significant (Supplementary Figure S1B).

HMGCS2 contributes to the regulation of intestinal cell differentiation, modulates the balance of cell differentiation and proliferation patterns, which are associated with different intestinal pathologies (e.g., colorectal cancer, inflammatory bowel disease and necrotizing enterocolitis) (Wang et al., 2017). Furthermore, it participates in the regulation and maintenance of intestinal epithelial homeostasis (Hegardt, 1999; Kim et al., 2019). HMGCS2 expression was increased in differentiated sections of the intestinal mucosa and disturbed expression of HMGCS2 in intestinal cells impairs cell differentiation (Wang et al., 2017). In addition, intestinal cell differentiation is enhanced by ketogenesis, which also inhibits abnormal cell growth. Whether the overexpression of HMGCS2 is a consequence of a reduced differentiation of epithelial cells or responds to other mechanisms is unknown. Here, contrary to what happens in WT mice, HMGCS2 levels are affected by a fat challenge in Dicer1 KO mice (Figure 2A), and this dysregulation deserves further investigation.

On the other hand, ACAT1 acts in the final stage of ketone breakdown (ketolysis) in the processing of lipids and is also able to catalyze the reverse chemical reaction, promoting the first step of ketogenesis (Kano et al., 1991). Here, the increase in *Acat1* gene expression observed in small intestine and intestinal organoids from Int-*Dicer1* KO mice compared to WT mice (Figures 2A,B), could corroborate the notion that miRNAs in enterocytes participate in the modulation of the ketogenesis process. Nevertheless, when compared to WT mice, *Acat1* gene expression findings in enterocytes from Int-*Dicer1* KO mice were not accompanied by increased protein levels (Figure 2C).

### *Olr1* and Intestinal Epithelial Differentiation Are Modulated by Intestinal miRNAs

The loss of *Dicer1* in the small intestine causes changes in permeability and intestinal epithelial differentiation (McKenna et al., 2010). In our study, OLR1 gene and protein expressions were increased in small intestine samples from Int-*Dicer1* KO mice compared to WT mice (Figures 2A,C). Enhanced intestinal permeability or incorrect intestinal differentiation can induce an inflammatory response in intestinal microcirculation, which may be accompanied by a rise in the expression of *Olr1* (Al-Banna and Lehmann, 2013). OLR1 has a structure capable of recognizing negatively charged substances, damaged cells, toxins, and bacteria (Wu et al., 2011; Al-Banna and Lehmann, 2013). Hence, OLR1 represents a therapeutic goal for modulating the inflammatory response in the intestinal microcirculation (Al-Banna and Lehmann, 2013). In agreement, and in contrast to what was observed in the intestinal tissue, OLR1 gene and protein expression in Int-Dicer1 KO mice was downregulated in intestinal organoids compared to WT (Figures 2B,D). To interpret these differences, we note that, although the intestinal organoids model approximates an *in vivo* situation better than single cell cultures. it lacks intestinal microbiota, lamina propria immune cells, and intestinal microcirculation (Angus et al., 2019).

**FIGURE 3:**
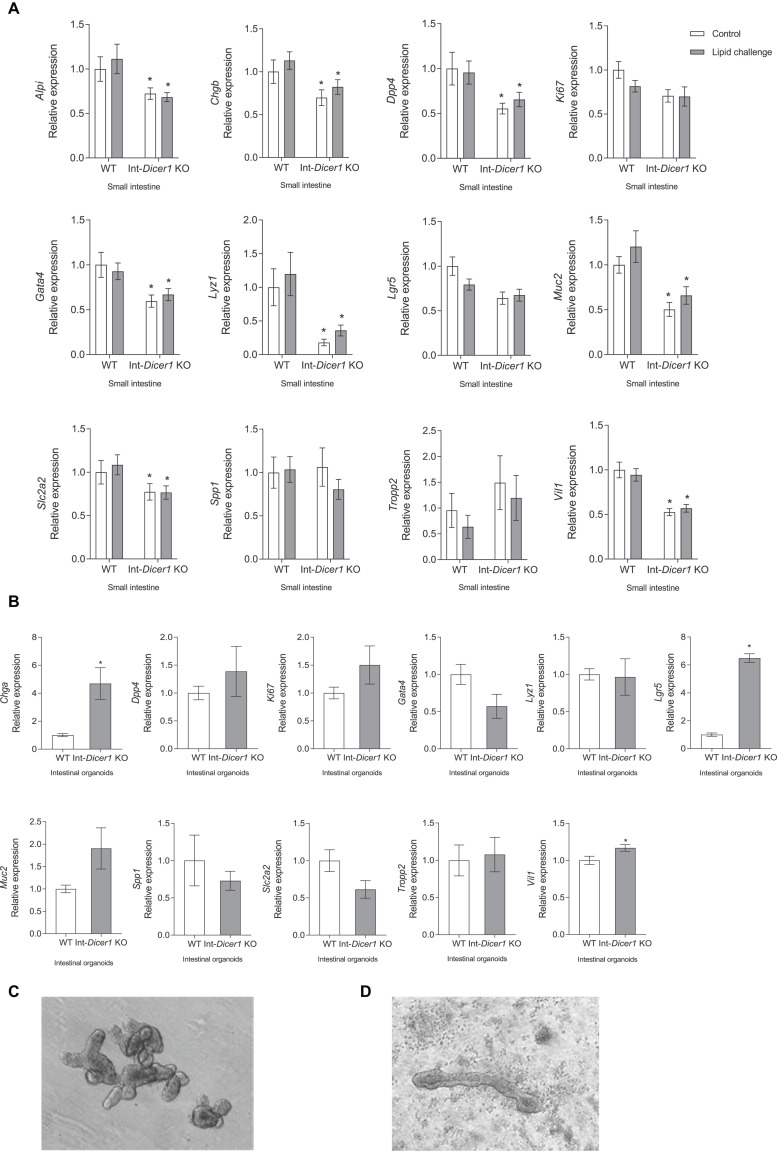
**(A)** Effect of an oral high-fat dietary challenge (lipid challenge) on the expression of *Alpi*, *Chgb*, *Dpp4*, *Ki67*, *Gata4*, *Lyz1*, *Lgr5*, *Muc2*, *Slc2a2*, *Spp1*, *Tropp2* and *Vill1*, in small intestine of Wild Type C57BL/6 (WT) and intestinal-specific *Dicer1* knockout (Int-*Dicer1* KO) mice. Data are means ± SEM. Comparison between groups by two-way ANOVA. *p < 0.05 compared to WT+H_2_O mice (*n* ≥ 23). **(B)** *Chga*, *Dpp4*, *Ki67*, *Gata4*, *Lyz1*, *Lgr5*, *Muc2*, *Slc2a2*, *Spp1*, *Tropp2* and *Vill1* gene expression in intestinal organoids isolated from WT and Int-*Dicer1* KO mice. Data are means ± SEM. Comparison between groups by two-tailed unpaired t-tests. *p < 0.05 compared to WT group (*n* = 9). **(C)** Light microscope visualization (10×) of mature intestinal organoids isolated from WT mice. **(D)** Light microscope visualization (10×) of mature intestinal organoids from Int-*Dicer1* KO mice.

We measured the levels of important proliferation/differentiation-related genes to assess if the aforementioned changes were associated with specific gene expression alterations. In small intestines, the expression of *Vil-1*, *Muc2*, *Chgb*, *Gata4*, *Alpi*, *Dpp4*, *Slc2a2* and *Lyz1* was significantly reduced in Int-*Dicer1* KO mice (Figure 3A). *Mucin 2* (*Muc2*) expression is frequently employed to identify variations in quantity functionality of intestinal goblet cells (Yu et al., 2016). *Chromogranin A (Chga)* is a marker of enteroendocrine cells (Andres et al., 2015). *Gata 4* is a marker of intestinal epithelial differentiation that is essential to preserve gut barrier function and mucosal integrity (Lepage et al., 2016). *Intestinal alkaline phosphatase (Alpi)* is a brush border enzyme that considerably diminishes the pro−inflammatory action of LPS (Parlato et al., 2018). *Dipeptidyl peptidase* (*Dpp4)* expression is increased in inflammatory bowel disease, atherosclerosis, obesity, and multiple sclerosis, implying its participation in the pathogenesis of inflammation (Zhou et al., 2019). *Solute carrier family 2 member 2 (Slc2a2)* is a β-cell glucose transporter necessary for standard glucose-stimulated insulin release (Novosadova et al., 2018). *Lysozyme 1* (*Lyz1)* is a marker of Paneth cells (Yu et al., 2016). Finally, *leucine-rich repeat-containing G-protein coupled receptor 5 (Lgr5)* stands as a marker of intestinal stem cells (Yu et al., 2016). The changes seen here suggest there is a different intestinal epithelium state between both murine models and a different proliferative rate, gut barrier function and mucosal integrity in Int-*Dicer1* KO mice. These data suggest that the lack of miRNAs impacts on intestinal development and permeability by influencing intestinal stem cells and proliferating transit amplifying cells, which give rise to distinct cell types (absorptive, goblet, and enteroendocrine cells).

Furthermore, *Vil1*, *Lgr5* and *Chga* expression was increased in Int-*Dicer1* KO mice compared to WT (Figure 3B). Intestinal organoids were used as a model for assessing the importance of miRNAs in intestinal epithelial development. Intestinal organoids derived from a relatively pure population of intact intestinal Lgr5+ stem cell-containing crypts (Angus et al., 2019) present a morphology that closely resembles the ones obtained from the WT mice used here (Figure 3C). However, a deviation from the expected intestinal organoid structure was seen for organoids derived from Int-*Dicer1* KO mice. Int-*Dicer1* KO organoids showed an unstructured morphology with elongated organoids (Figure 3D), suggesting miRNAs play a relevant role in intestinal epithelial development.

### miRNAs Candidates to Target Intestinal *Hmgcs2*, *Acat1* or *Orl1*

**FIGURE 4:**
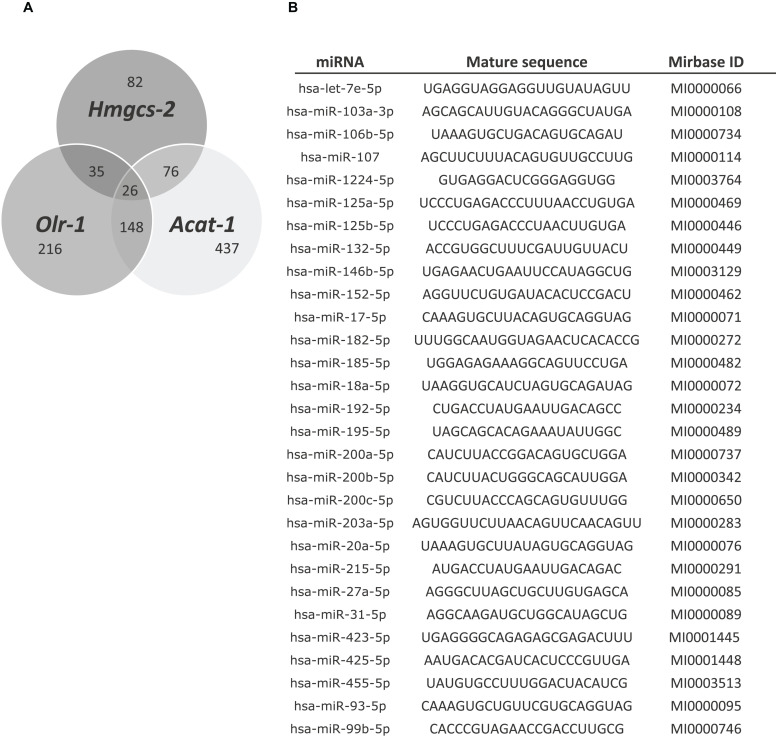
**(A)** PITA and TargetScan algorithms were run to predict 26 common miRNAs putatively regulating *Hmgcs-2*, *Acat-1 and Olr-1*. **(B)** miRNAs selected comparing the bioinformatic analysis with the list of 173 miRNAs expressed in intestinal epithelium.

First, the possible miRNAs regulating these genes were bioinformatically analyzed as different miRNAs can target one gene. To do this, PITA and TargetScan were searched for miRNA-gene interactions. Twenty-six putative miRNAs capable of targeting *Hmgcs2*, *Acat1* and *Olr1* simultaneously, and 76 capable of targeting *Hmgcs2* and *Acat1* simultaneously were identified (Figure 4A). These results were compared with the highly enriched miRNAs expressed in intestinal epithelium (Supplementary Figure S2). The search was then centered in miRNAs that were both expressed in the small intestine and predicted by bioinformatic analysis, and 29 miRNAs were finally selected (Figure 4B).

**FIGURE 5:**
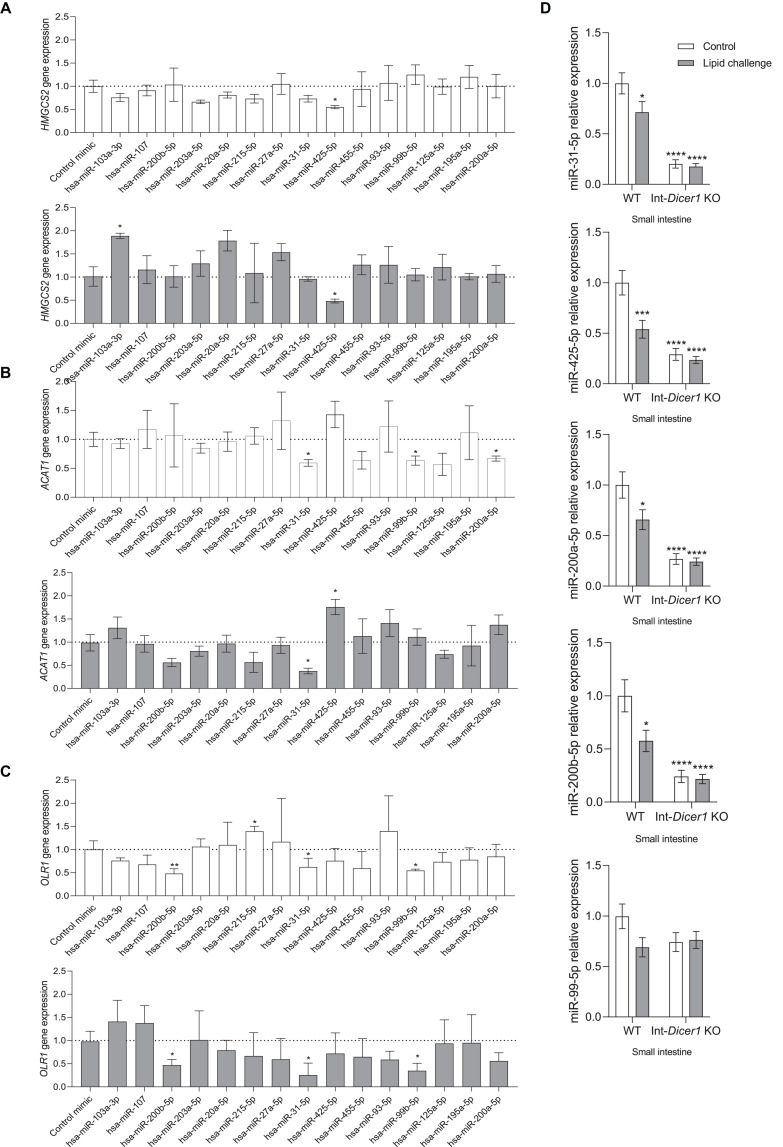
Effects of several hsa-miRs on the expression levels of **(A)** *Hmgcs2*, **(B)** *Acat1* and **(C)** *Olr1*, in Caco-2 cells transfected for 24 or 48 h. Data are means ± SEM. Comparison between groups by two-tailed unpaired *t*-tests. *p < 0.05 compared to control group. **p < 0.001 compared to control group (*n* = 4). **(D)** Effects of an oral high-fat dietary challenge (lipid challenge) on the expression of selected miRNAs, in small intestine of Wild Type C57BL/6 (WT) and intestinal-specific *Dicer1* knockout (Int-*Dicer1* KO) mice. Data are means ± SEM. Comparison between groups by two-way ANOVA. *p < 0.05 compared to WT+H_2_O mice (*n* ≥ 23).

Screening and validation *in vitro* lessens misleading estimations; thus, transfection with miRNA mimics for 24 or 48 h was performed in Caco-2 cells, and *Hmgcs2*, *Acat1* and *Olr1* gene expression levels were measured by RT-qPCR (Figure 5). A negative mimic miRNA with no known target served as a control. Many of these miRNA-target interactions were not validated (Supplementary Figure S3).

### Validation Confirms Five Selected miRNAs

Our findings provide the first evidence that miR-425-5p negatively modulates the gene expression of *Hmgcs2* in Caco-2 cells (24 and 48 h) (Figure 5A). The miR-425 family (including miR-425-3p and miR-425-5p) participates in biological processes occurring in the gastrointestinal tract, among other tissues, and its abnormal expression is associated with the progression of several diseases (Lu et al., 2019). Interestingly, the increase in miR-425 levels is useful for fat production in muscle cells and adipocytes (Qi et al., 2019). According to the results shown here, miR-425-5p appears to be important in the regulation of lipid metabolism, ketogenesis, and intestinal differentiation in enterocytes.

Even though miR-31-5p, miR-99b-5p and miR-200a-5p exhibited significant *Acat1* gene expression downregulation capacity, 24 h after transfection, only the former exerted this effect also after 48 h (Figure 5B). MiR-31-5p, miR-99b-5p and miR-200b-5p downregulated *Olr1* levels in Caco-2 culture cells, at 24 and 48 h (Figure 5C). To determine whether the five above-mentioned miRNAs were modulated in the intestines of Int-*Dicer1* KO mice, we evaluated their expression by RT-qPCR (Figure 5D). We found that miR-425-5p, miR-31-5p, miR-200a-5p and miR-200b-5p were dramatically repressed in the small intestine of Int-*Dicer1* KO mice, which is in accordance with the depression seen for *Hmgcs2*, *Acat1* and *Olr1*. Related studies have found that levels of miR-31-5p were enhanced in inflamed mucosa from patients with ulcerative colitis and Chron‘s disease, and play a fundamental role in epithelial cell regeneration (Gupta et al., 2019; Tian et al., 2019). Mir-99b-5p is a marker of inflammation in tissues other than the intestine (Hildebrand et al., 2018) and miR-200b-5p was linked with the heightened expression of interleukin 8 (IL-8), CXCL2, IL-1β, tumor necrosis factor alpha (TNF-α), and IL-6 in upper genital tract disease (Yeruva et al., 2017). Further, increased intestinal permeability or incorrect intestinal differentiation can induce an inflammatory response in the intestinal microcirculation, which may be accompanied by a rise in the expression of *Olr1* (Al-Banna and Lehmann, 2013). Hence, results suggest that miR-31-5p, miR-99b-5p, and miR-200b-5p negatively modulate the gene expression of *Olr1* and could represent a novel therapeutic target against intestinal inflammatory processes.

## Conclusion

Loss of Dicer1 in mouse intestinal epithelial cells unveiled three miRNA-regulated genes, i.e., *Hmgcs2*, *Acat1* and *Orl1*. Intestinal organoids isolated from these mice exhibited different morphological features from those of wild type mice. Although further validation is needed, we provide evidence that miR-425-5p, miR-31-5p, miR-99b-5p, miR-200a-5p and miR-200b-5p can function as major regulators of lipid metabolism-related genes in the intestine. Moreover, in view of the results, we hypothesize that miR-425-5p is involved in the expression level changes in ketogenesis-related genes observed after the dietary lipid challenge. Further insight on the role of miRNAs as modulators of the molecular actions in response to dietary cholesterol, lipid homeostasis and intestinal ketogenesis may reveal other potential therapeutic targets involved in the regulation of lipid and cholesterol metabolism, as well as ketogenesis, in the gut. In this sense, more *in vivo* research is needed before firm conclusions can be drawn in terms of therapeutic approaches to modulate intestinal miRNAs in health and disease.

## Materials and Methods

### Animals

Animal practices were performed in agreement with the guidelines of the European Communities Directive 86/609/EEC controlling animal research and was approved (Proex 281/15 and Proex 282/15) by the Animal Ethics Committee of the Ramón y Cajal Hospital (Madrid, Spain). Int-*Dicer1* KO and wild type (WT) littermates [fl/fl, tg(-)] C57BL/6J mice, 8–10 weeks old, were used in all experiments. Mice were housed in a standard animal facility and maintained in a temperature- (25 ± 2°C) and light- (12 h light–dark cycles) controlled room. Food and water were available *ad libitum*.

### Study Design

Male and female C57BL/6 WT littermates and Int-*Dicer1* KO mice were distributed into two experimental groups. Mice received (by oral gavage) either a high-fat dietary challenge (lipid challenge), consisting on the administration of 250 μL olive oil enriched with 40 mg of cholesterol, or water (controls). Two hours after administration, mice were anesthetized with ketamine/xylazine, sacrificed by exsanguination and perfused with phosphate-buffered saline (PBS).

### Sample Collection

Blood samples were immediately collected in EDTA tubes and centrifuged at 1,500 × *g*, for 15 min, at 4°C, to obtain plasma. In addition, small intestine samples, including scraped mucosa, were collected and immediately frozen in liquid nitrogen. All samples were stored at −80°C.

### Isolation of Mouse Small-Intestinal Crypts and Organoid Culture

Intestinal crypts were isolated from the small intestine of C57BL/6 WT [fl/fl, tg(-)] mice or littermates from intestinal-specific *Dicer1* knockout (Int-*Dicer1* KO) mice, according to Stemcell’s recommended protocol [Technical Bulletin: Intestinal Epithelial Organoid Culture with IntestiCult^TM^ Organoid Growth Medium (Mouse)]. Intestinal crypts were plated in 24-well tissue culture plates (Corning, NY, United States) (200 crypts per well) and incubated at 37°C and 5% CO_2_ until passage.

### Caco-2 Culture Cells and Transfection

Caco-2 cells were acquired from the American Type Culture Collection (ATCC). Cells were maintained in DMEM medium (Invitrogen, Carlsbad, CA, United States) supplemented with 10% heat-inactivated fetal bovine serum (FBS, Invitrogen, CA, United States), 100 IU/mL penicillin and 100 μg/mL streptomycin (Invitrogen, Carlsbad, CA, United States) at 37°C in a humidified incubator with 5% CO_2_.

All miRNAs were synthesized by Cultek Molecular Bioline (Madrid, Spain). Caco-2 cells were transfected using Lipofectamine 2000 (Invitrogen, Carlsbad, CA, United States), according to the manufacturer’s recommendations, and 20 μM miRNAs diluted in Opti-MEM^®^ (Invitrogen, Carlsbad, CA, United States). For conventional aqueous transfection, 4.5 × 10^4^ cells per well were cultured in 24-well plates, for 24 h. Then, Lipofectamine 2000-miRNA complexes diluted (1:5) in culture medium were added. Medium was replaced 24 or 48 h after the transfection.

### Intestinal Organoids Treatment

Organoids were incubated in 24-well tissue culture plates and treated with postprandial micelles (including olive oil and cholesterol, PPM) or DMEM (control group), for 24 h. Artificial micelles were prepared according to a previously described method (Briand et al., 2016). The final composition of this micelles was 0.6 mM oleic acid (OA), 0.2 mM L-α-lysophosphatidylcholine, 0.05 mM cholesterol, 0.2 mM 2-monooleylglycerol, 2 mM taurocholate. The emulsion was sonicated twice for 30 min. The micelle solution was incubated for 2 h at 37°C. Each compound was added to the micelle solution and incubated for a further 2 h at 37°C.

### RNA Isolation and qRT-PCR

Total RNA was extracted from tissue samples with TRIzol reagent (Invitrogen, Carlsbad, CA, United States), according to the manufacturer’s instructions. Total RNA was isolated using the miRNeasy Mini Kit (Qiagen). cDNA was synthesized using Taqman Reverse Transcription Reagents (Applied Biosystems). qRT-PCR was performed on a 7900 HT Fast Real-Time PCR System (Applied Biosytems), using the miScript SYBR Green PCR kit (Qiagen). mRNA levels were normalized to those of the housekeeping gene *beta-Actin* and *Gapdh*. Relative expressions were calculated by the comparative threshold cycle method and presented as a relative expression ratio (2-ΔΔ threshold cycle). For miRNA quantification, total RNA was reverse-transcribed using the miScript II Reverse Transcription Kit (Qiagen). Specific primers for each miRNA (miScript Primer Assay) were also obtained from Qiagen. MiRNA levels were normalized to that of the housekeeping RNU6 (U6). List of specific oligos for mRNA genes are described in Supplementary Figure 4.

### Western Blotting

Proteins were separated on SDS-PAGE gels under reducing conditions and transferred to nitrocellulose membranes (Bio-Rad, Hercules, CA, United States). Membranes were blocked for 1 h with 5% (w/v) bovine serum albumin (BSA) as a blocking agent (Sigma, Madrid, Spain) in PBS with Tween 20 (PBST; 1% PBS, 0.1% Tween20; v/v) at room temperature. After washing, membranes were probed overnight, at 4°C, with appropriate primary antibodies: HMGCS2 1:1000 (D3U1A, Cell signaling technology), ACAT1 1:1000 (CSB-PA001134LA01HU, Cusabio technology), DICER1 1:2000 (A301-936A, Bethyl laboratories) and OLR1 (CSB-PA016331LA01HU, Cusabio technology). After washing, membranes were incubated for 1 h with peroxidase-conjugated rabbit or mouse anti-goat IgG secondary antibody (1:10,000). For detection, ECL Advance Western Blotting Detection kit (Amersham Bioscience, Amersham, United Kingdom) was used. Blots were probed with rabbit monoclonal anti-Actin antibody (1:10,000, Abcam, Cambridge, United Kingdom) or rabbit monoclonal anti-HSP90 antibody (1:10,000, Abcam, Cambridge, United Kingdom) as internal control, to normalize between gels. Quantification was expressed as the percentage of relative protein expression (protein/Actin or HSP90) vs. control group.

### Gene Expression Profiling of Small Intestine by RT2-PCR Array

Expression of 83 genes involved in lipid and cholesterol metabolism was analyzed by PCR array using a 384-well 96 × 4 Mouse Lipoprotein & Cholesterol Metabolism RT2 Profiler PCR Array Kit (Qiagen) and a 7900 HT Fast Real-Time PCR Software (Applied Biosystems). Gene expression was normalized to the mean of all house-keeping genes in the array.

### Bioinformatic Analysis to Identify miRNAs-Gene Interactions

Two different algorithms were implemented simultaneously in order to identify miRNA-gene interactions: PITA and TargetScan. TargetScan (Friedman et al., 2009) uses the degree of sequence complementarity as the primary key parameter to identify miRNA-mRNA interactions. PITA (Kertesz et al., 2007) utilizes thermodynamics as the main criterion. A prediction was considered valid whenever it co-occurred in at least two algorithms.

### Statistical Analysis

Data are shown as means ± standard error of the mean (SEM). Statistical analyses consisted of two-way analysis of variance (ANOVA) (genotype × lipid challenge), followed by Tukey’s *post-hoc* tests, or two-tailed unpaired *t*-tests. A significance level of *p* < 0.05 was applied to all statistical analyses. GraphPad Prism 8 (version 8.3.0; Graph Pad Software Inc., San Diego, CA, United States) was used for all statistical analyses.

## Data Availability Statement

All datasets presented in this study are included in the article/Supplementary Material.

## Ethics Statement

The animal study was reviewed and approved by Animal research was approved (Proex 281/15 and Proex 282/15) by the Animal Ethics Committee of the Ramón y Cajal Hospital (Madrid, Spain).

## Author Contributions

AD: idea and coordination of work. MR-R: bioinformatic analysis, mathematical analysis, statistical treatment of the data and wrote the manuscript, prepared figures and illustrations. MR-R, JG-Z, MCL, OB, DS-L, MC, MJL, JT-C, and AD: performed experiments. JG-Z and AO: veterinary work. JT-C, FV, and AD: edited the manuscript. MR-R, AD, FV, OB, and JM: administrative support and discussion. All the authors have read and approved the final manuscript.

## Conflict of Interest

The authors declare that the research was conducted in the absence of any commercial or financial relationships that could be construed as a potential conflict of interest.

## Supplementary Material

FIGURE S1 Effect of an oral high-fat dietary challenge (lipid challenge) on the expression of **(A)** *Abca2*, *Lipe*, *Nrob2*, and *Prkaa* (validation assays were not performed), **(B)** *Bdh1* and **(C)** *Hmgcl*, in small intestine of Wild Type C57BL/6 (WT) and intestinal-specific *Dicer1* knockout (Int-*Dicer1* KO) mice. Data are means ± SEM. Comparison between groups by two-way ANOVA **(A)** or two-tailed unpaired *t*-tests (B,C). ^∗^*p* < 0.05 compared to WT+H_2_O mice or WT (*n* ≥ 23 per group).

FIGURE S2 List of 173 miRNAs expressed in intestinal epithelium and their relative mean expression.

FIGURE S3 Effect of selected miRNAs on relative gene expression of *Hmgcs-2*, *Acat-1* and *Olr-1* in Caco-2 cells after 24 and 48 h. Data are means relative to control.

FIGURE S4 Sequences of quantitative RT-PCR Primers.
