# Supplementary material for: Intestinal Lipid Metabolism Genes Regulated by miRNAs
Source: Front Genet. 2020 Jul 10;11:707. doi: 10.3389/fgene.2020.00707 (PMC7366872; doi:10.3389/fgene.2020.00707)
Supplement: FIGURE S1 — Effect of an oral high-fat dietary challenge (lipid challenge) on the expression of (A) Abca2, Lipe, Nrob2, and Prkaa (validation assays were not performed), (B) Bdh1 and (C) Hmgcl, in small intestine of Wild Type C57BL/6 (WT) and intestinal-specific Dicer1 knockout (Int-Dicer1 KO) mice. Data are means ± SEM. Comparison between groups by two-way ANOVA (A) or two-tailed unpaired t-tests (B,C). ∗p < 0.05 compared to WT+H2O mice or WT (n ≥ 23 per group). [file Data_Sheet_1.PDF]

Figure Supplementary 1

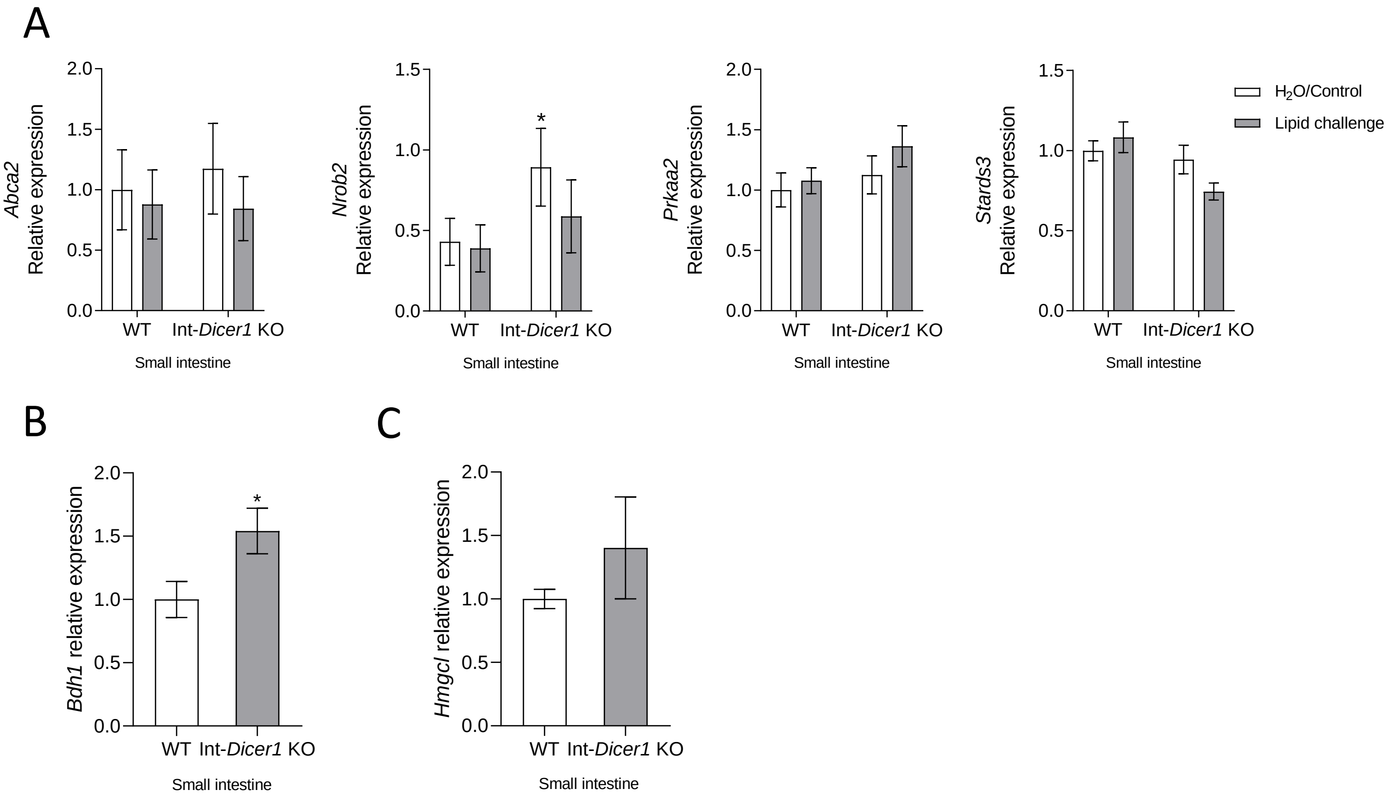

Figure Supplementary 2

MiRNAs	Mean relative expression	MiRNAs	Mean relative expression	MiRNAs	Mean relative expression	MiRNAs	Mean relative expression	MiRNAs	Mean relative expression
**mmu-let-7f-5p**	254706.61	**mmu-miR-152-3p**	902.10	**mmu-miR-125b-5p**	140.83	**mmu-miR-17-5p**	47.88	**mmu-miR-139-5p**	24.17
**mmu-let-7c-5p**	168880.26	**mmu-miR-200a-3p**	848.37	**mmu-miR-1981-5p**	140.38	**mmu-miR-30a-3p**	47.37	**mmu-miR-100-5p**	23.77
**mmu-miR-192-5p**	137628.33	**mmu-miR-148a-3p**	842.49	**mmu-miR-30c-5p**	131.17	**mmu-miR-7a-5p**	47.06	**mmu-miR-30c-2-3p**	23.49
**mmu-miR-215-5p**	100798.29	**mmu-miR-92a-3p**	821.22	**mmu-miR-30e-5p**	128.30	**mmu-miR-130b-3p**	46.65	**mmu-miR-345-5p**	22.02
**mmu-let-7b-5p**	97417.26	**mmu-miR-31-5p**	729.37	**mmu-miR-143-5p**	127.64	**mmu-miR-676-3p**	45.76	**mmu-miR-20a-5p**	21.95
**mmu-let-7a-5p**	94577.10	**mmu-miR-30d-5p**	722.38	**mmu-miR-16-5p**	116.94	**mmu-miR-669c-5p**	45.28	**mmu-miR-28a-5p**	21.48
**mmu-let-7g-5p**	13043.17	**mmu-miR-215-3p**	632.14	**mmu-miR-27a-3p**	113.73	**mmu-miR-25-5p**	43.19	**mmu-miR-423-3p**	21.09
**mmu-let-7d-5p**	12023.71	**mmu-miR-181a-5p**	630.08	**mmu-miR-674-5p**	108.95	**mmu-miR-532-5p**	42.37	**mmu-miR-194-1-3p**	20.63
**mmu-miR-1a-3p**	12016.98	**mmu-miR-24-3p**	613.17	**mmu-miR-99b-5p**	108.60	**mmu-miR-331-3p**	41.66	**mmu-miR-99a-5p**	20.47
**mmu-miR-140-3p**	7964.26	**mmu-miR-101b-3p**	573.25	**mmu-miR-451a**	105.77	**mmu-miR-30b-5p**	41.57	**mmu-miR-214-3p**	19.19
**mmu-miR-21a-5p**	7322.40	**mmu-miR-200b-3p**	558.54	**mmu-miR-132-3p**	86.95	**mmu-miR-365-3p**	41.57	**mmu-miR-532-3p**	18.71
**mmu-miR-29a-3p**	6794.23	**mmu-miR-200c-3p**	487.95	**mmu-miR-10b-5p**	84.85	**mmu-miR-34c-5p**	40.91	**mmu-miR-455-3p**	17.80
**mmu-let-7e-5p**	6166.63	**mmu-miR-181b-5p**	452.72	**mmu-miR-3095-3p**	80.78	**mmu-let-7d-3p**	40.04	**mmu-miR-23b-5p**	17.78
**mmu-miR-103-3p**	6130.57	**mmu-miR-429-3p**	445.08	**mmu-miR-15a-5p**	80.26	**mmu-miR-222-3p**	37.89	**mmu-miR-22-5p**	17.55
**mmu-miR-143-3p**	5733.77	**mmu-miR-30a-5p**	442.47	**mmu-miR-146a-5p**	79.90	**mmu-miR-216b-5p**	37.78	**mmu-miR-382-5p**	16.61
**mmu-miR-191-5p**	4898.57	**mmu-miR-23b-3p**	404.43	**mmu-miR-3473b**	79.65	**mmu-miR-196a-5p**	37.76	**mmu-miR-1983**	16.48
**mmu-miR-10a-5p**	3910.55	**mmu-miR-29c-3p**	387.89	**mmu-miR-106b-5p**	78.83	**mmu-miR-19b-3p**	36.48	**mmu-miR-186-5p**	16.28
**mmu-miR-423-5p**	3396.07	**mmu-miR-22-3p**	376.71	**mmu-miR-195a-5p**	73.99	**mmu-miR-148b-3p**	36.29	**mmu-miR-106b-3p**	15.96
**mmu-miR-375-3p**	3385.89	**mmu-miR-203-3p**	376.57	**mmu-miR-130a-3p**	72.45	**mmu-miR-425-5p**	34.86	**mmu-miR-322-5p**	15.49
**mmu-miR-199a-3p**	2924.50	**mmu-miR-181d-5p**	367.95	**mmu-miR-182-5p**	70.62	**mmu-miR-181c-5p**	32.10	**mmu-miR-30e-3p**	15.25
**mmu-miR-199b-3p**	2924.43	**mmu-miR-98-5p**	255.46	**mmu-miR-345-3p**	70.31	**mmu-miR-363-5p**	31.32	**mmu-miR-223-3p**	14.83
**mmu-miR-107-3p**	2780.32	**mmu-miR-128-3p**	221.52	**mmu-miR-217-5p**	68.03	**mmu-miR-138-5p**	31.27	**mmu-miR-708-5p**	14.21
**mmu-miR-194-5p**	2653.02	**mmu-miR-342-3p**	218.66	**mmu-miR-322-3p**	65.30	**mmu-miR-28a-3p**	31.01	**mmu-miR-484**	14.10
**mmu-let-7i-5p**	2567.94	**mmu-miR-340-5p**	215.97	**mmu-miR-374b-5p**	63.17	**mmu-miR-434-3p**	30.87	**mmu-miR-186-3p**	13.95
**mmu-miR-145a-5p**	2506.33	**mmu-miR-221-3p**	212.18	**mmu-miR-3473e**	62.09	**mmu-miR-17-3p**	30.19	**mmu-miR-192-3p**	13.72
**mmu-miR-320-3p**	2500.91	**mmu-miR-378c**	211.81	**mmu-miR-150-5p**	59.80	**mmu-miR-221-5p**	29.75	**mmu-miR-511-3p**	13.28
**mmu-miR-378a-3p**	2378.24	**mmu-miR-125a-5p**	205.19	**mmu-miR-133a-3p**	55.62	**mmu-miR-21a-3p**	29.21	**mmu-miR-27b-5p**	13.27
**mmu-miR-26b-5p**	2045.15	**mmu-miR-200b-5p**	192.10	**mmu-miR-193a-3p**	55.13	**mmu-miR-31-3p**	28.89	**mmu-miR-96-5p**	13.07
**mmu-miR-26a-5p**	1806.88	**mmu-miR-33-5p**	192.01	**mmu-miR-141-3p**	52.52	**mmu-miR-126a-5p**	27.84	**mmu-miR-877-5p**	12.90
**mmu-miR-25-3p**	1715.29	**mmu-miR-15b-5p**	188.01	**mmu-miR-145a-3p**	52.06	**mmu-miR-350-3p**	27.54	**mmu-miR-341-3p**	12.18
**mmu-miR-1839-5p**	1646.61	**mmu-miR-93-5p**	181.62	**mmu-miR-497-5p**	51.92	**mmu-miR-151-3p**	26.37	**mmu-miR-541-5p**	12.05
**mmu-miR-185-5p**	1532.69	**mmu-miR-27b-3p**	175.27	**mmu-miR-129-2-3p**	51.81	**mmu-miR-3107-5p**	25.57	**mmu-miR-7a-1-3p**	11.25
**mmu-miR-142-5p**	1300.87	**mmu-miR-744-5p**	172.37	**mmu-miR-142-3p**	49.12	**mmu-miR-486-5p**	25.50	**mmu-miR-199b-5p**	11.17
**mmu-miR-101a-3p**	927.38	**mmu-miR-29b-3p**	162.25	**mmu-miR-598-3p**	48.47	**mmu-miR-183-5p**	24.96		
**mmu-miR-23a-3p**	917.73	**mmu-miR-146b-5p**	159.12	**mmu-miR-872-5p**	48.30	**mmu-miR-151-5p**	24.20		

Figure Supplementary 3

	Caco2- 24h			Caco2-48h		
	Hmgcs2 individual score	Acat1 individual score	Olr1 individual score	Hmgcs2 individual score	Acat1 individual score	Olr1 individual score
**Control mimic**	1	1	1	1	1	1
**hsa-let-7e-5p**	3.67	2.01	2.81	9.17	1.89	1.3
**hsa-miR-106b-5p**	2.66	1.05	1.88	6.94	1.01	1
**hsa-miR-1224-5p**	3.06	1.27	1.69	12.67	0.89	1.4
**hsa-miR-125a-5p**	3.22	1.11	1.37	10.08	0.78	0.96
**hsa-miR-125b-5p**	3.07	1.3	1.26	10.94	1.24	1.97
**hsa-miR-132-5p**	3.1	1.65	2.27	10.52	1.23	2.09
**hsa-miR-146b-5p**	3.25	1.04	1.04	12.32	1.33	1.26
**hsa-miR-152-5p**	2.66	1.02	1.76	9.98	0.92	1.05
**hsa-miR-17-5p**	3.18	1.04	1.08	9.21	1.76	1.68
**hsa-miR-182-5p**	3.41	1.53	1.67	13.53	2.38	2.47
**hsa-miR-185-5p**	3.42	1.89	1.94	13.56	2.53	2.69
**hsa-miR-18a-5p**	4.44	2.73	2.69	13.23	4.12	2.62
**hsa-miR-192-5p**	2.56	1.42	1.43	13.24	2.1	1.42
**hsa-miR-200c-5p**	2.62	1.31	1.51	11.28	1.52	1.17
**hsa-miR-423-5p**	2.5	3.53	4.96	1.66	1.71	1.81

Figure Supplementary 4

Gene	Name	Primer Sequence
Hmgcs2	Hmgcs2-FW	GAAGAGAGCGATGCAGGAAAC
	Hmgcs2-REV	GTCCACATATTGGGCTGGAAA
Nr0b2	Nr0b2-FW	GGGAGCAGGAAGCTGTACTTTCAA
	Nr0b2-REV	ATGAGTCCCCTCCAGCAAGACTAA
Olr1	Olr1-FW	CCAGCCTCAGCATCTCAAGTTACA
	Olr1-REV	GTTAAGAGCACTGCTTGCCTTTCC
Prkaa2	Prkaa2-FW	CAGGCCATAAAGTGGCAGTTA
	Prkaa2-REV	AAAAGTCTGTCGGAGTGCTGA
Acat1	Soat1-FW	TACCGAGACAACTACCCAAGGACT
	Soat1-REV	GCACTGAAGGGCTCCTGTTTGATA
Dicer1	Dicer1-FW	TCACTAGGGCACTTGCAATGGAAC
	Dicer1-REV	ACTGCATCTCAGGCTCATTTCGTC
Abca1	Abca1-FW	GGATACAATCGATGCGTCTGACCT
	Abca1-REV	ACGGATGACATTGAGCACTGGT
Abca2	Abca2-FW	CGCAGTTTGATGCCCTGTTTGA
	Abca2-REV	AGAAAGATGAAGGCAGGGTACCCA
Apoa4	Apoa4-FW	TGGGGATGCTAGTACGTATGCTGA
	Apoa4-REV	TGCTCCTGCAACTTCTGCATGT
Apob	Apob-FW	TCTTTCAGGATCCCTCACTTCCCA
	Apob-REV	TAAATGCTCCCCAGTGACACCTCT
Apoc3	Apoc3-FW	TAAGTAGCGTGCAGGAGTCCGATA
	Apoc3-REV	ATAGCTGGAGTTGGTTGGTCCTCA
Cnbp	Cnbp-FW	GGGTTCCAGTTTGTTTCCTCGTCT
	Cnbp-REV	TCCTTGCAGTCCTTGGCAATGT
Crp	Crp-FW	CGGAGATGTGAACATGTGGGACTT
	Crp-REV	GTGCCCGCCAGTTCAAAACATT
Cyb5r3	Cyb5r3-FW	GACGGTGAAGTCTGTAGGCATGAT
	Cyb5r3-REV	CGAGCAGAATGTTCGTTCCTCAGT
Lep	Lep-FW	AAGCAGTGCCTATCCAGAAAGTCC
	Lep-REV	ATAGACTGCCAGAGTCTGGTCCAT
Lipe	Lipe-FW	TGTGTTAGAGGGAGGGTGAAGGAT
	Lipe-REV	GGTGCCTGGTACACTCCAAATGTT
Osbpl1a	Osbpl1a-FW	AGACCCTGCCACTTTTGATGCT
	Osbpl1a-REV	TCGGAATCTGGCACTGGCATTT
Stard3	Stard3-FW	AGGAAGTAATCCTGCAGCCTGAGA
	Stard3-REV	CCGCTCAATACGTCGGACATTCA
Hmgcl	HMGCL-FW	GAAGCTGGGGACTTCATCTGTCAA
	HMGCL-REV	ATACACCCAATTCCCTCCGTTCTG
Bdh1	BDH1-FW	GTGCACTGTTCTAGCTCCTGTCTT
	BDH1-REV	CAGGCTCTGCAGTACAAATGCATC
Lgr5	Lgr5-FW	CGTCCCACTTCCTTCCTGTC
	Lgr5-REV	TATGCTGGCGTGGGTAAAGG
Ki67	ki67-FW	ACCATCATTGACCGCTCCTT
	ki67-REV	TATCTGCAGAAAGGCCCTTGG
Lyz1	Lyz1-FW	GAGACCGAAGCACCGACTATG
	Lyz1-REV	CGGTTTTGACATTGTGTTCGC
Vil1	Vil1-FW	GGAGGATCGAGGCTATGCAG
	Vil1-REV	GAGAGTGCTGCTGGTCTTGT
Alpi	Alpi-FW	ATGATGCCAACCGAAACCCC
	Alpi-REV	GCGTGTCCTTCTCATTGGTAA
Muc2	Muc2-FW	CCTGAAGACTGTCGTGCTGT
	Muc2-REV	GGGTAGGGTCACCTCCATC
Chga	Chga-FW	CCAAGGTGATGAAGTGCGTC
	Chga-REV	GGTGTCGCAGGATAGAGAGGA
Chgb	Chgb-FW	CAGACGAAGGTAGGCAATGAG
	Chgb-REV	GGTTGGAGGGACGGAGACT
Gata4	Gata4-FW	TCAACCGGCCCCTCATTAAG
	Gata4-REV	GTGGTGGTAGTCTGGCAGT
Dpp4	Dpp4-FW	CAGCTCATCCTCTAGTGCGG
	Dpp4-REV	GTAGCCCACACCACATCACA
Slc2a2	Slc2a2-FW	ATCACCGGAACCTTGGCTTT
	Slc2a2-REV	CAGCTTTCCGGTCATCCAGT
Tropp2	Trop2-FW	CACTCTGACCTAGACTCCGAG
	Trop2-REV	CGAAGCTCTATCTGAATGGTGG
Spp1	Spp1-FW	AGAGCGGTGAGTCTAAGGAGT
	Spp1-REV	TGCCCTTTCCGTTGTTGTCC
